# P2Y12 receptor gene polymorphisms are associated with epilepsy

**DOI:** 10.1007/s11302-022-09848-4

**Published:** 2022-02-17

**Authors:** Qi Wang, Nan-Rui Shi, Peng Lv, Juan Liu, Ji-Zhou Zhang, Bin-Lu Deng, Yan-Qin Zuo, Jie Yang, Xin Wang, Xiang Chen, Xiu-Min Hu, Ting-Ting Liu, Jie Liu

**Affiliations:** 1 School of Clinical Medicine, Southwest Medical University, 646000 Luzhou, China; 2 School of Acupuncture and Tuina, Chengdu University of Traditional Medicine, Chengdu, 610075 China; 3 School of Sports Medicine and Health, Sports Medicine Key Laboratory of Sichuan Province, Chengdu Sport University, Chengdu, 610041 China; 4 Department of Neurology, Sichuan Provincial People’s Hospital, University of Electronic Science and Technology of China, Chengdu, 610072 China; 5 Sichuan Academy of Medical Sciences &, Sichuan Provincial People’s Hospital, Chengdu, 610072 China

**Keywords:** P2Y12 receptor, Single-nucleotide polymorphism, Epilepsy

## Abstract

The basic research indicated that microglial P2Y12 receptors (*P2Y12Rs*) are involved in the pathophysiology of epilepsy through regulated microglial-neuronal interactions, aberrant neurogenesis, or immature neuronal projections. However, whether the clinic case of epilepsy would be associated with *P2Y12* receptor gene polymorphisms is presented with few data. In our study, a total of 176 patients with epilepsy and 50 healthy controls were enrolled. Two single-nucleotide polymorphisms, namely rs1491974 and rs6798347, were selected for analysis. The results revealed that carriers of the G allele of rs1491974 G>A or rs6798347 G>A may be associated with an increased risk of epilepsy (OR = 0.576, 95% CI = 0.368–0.901, *p* = 0.015; OR = 0.603, 95% CI = 0.367–0.988, *p* = 0.043). Interestingly, we found that the rs1491974 G>A genotype and allele frequencies have only a significant difference in female instead of male case (*p* = 0.004 for genotype; *p* = 0.001 for allele). The subgroup analysis demonstrated that individuals with the rs1491974 G>A genotype might have more frequent seizure (OR = 0.476, 95% CI = 0.255–0.890; *p* = 0.019). These data implied that both rs1491974 and rs6798347 polymorphisms of *P2Y12R* would be able to play import roles in epilepsy susceptibility, whereas the rs1491974 polymorphism may be specifically related to seizure frequency.

## Introduction

Epilepsy is one of the most common neurological disorders which affects over 70 million people globally and imposes a considerable socio-economic burden [1-3]. The etiology of epilepsy is diverse and remains elusive [4]. Among various factors, genetic mutations, such as single-nucleotide polymorphisms (SNPs), are a common cause of epilepsy and are generally associated with ion channels, neuronal receptors, transcription factors, and enzymes [5-8. Accumulating evidence has shown that purinergic signaling SNPs, including adenosine kinase SNPs, adenosine A1 receptor SNPs, and adenosine A2A receptor SNPs, are implicated in the pathogenesis mechanism of epilepsy [9-11]. However, purinergic signaling is a big family. It includes purines (ATP, ADP, AMP, adenosine), enzymes (CD39, CD73), and purinergic receptors (four P1 receptors, seven P2X receptors, and eight P2Y receptors). Moreover, purinergic signaling has been recognized as promising targets for the treatment of various central nervous system (CNS) diseases [12-16]. The role of these purinergic signaling SNPs in epilepsy, especially P2Y12 receptor (*P2Y12R*), has not been investigated yet.

The P2Y12 receptor is part of the metabotropic (P2Y) receptor family with exclusive expression in the microglia of the CNS [17,18], and it is essential for brain homeostasis including synaptic plasticity [19], vascular repair [20], and chemotaxis and motility of microglia [21,22]. Several studies revealed the *P2Y12R* plays a pivotal role in the pathophysiology of several CNS disorders, including seizures [23-27]. Eyo and colleagues reported that seizure outcome worsened in *P2Y12R* knockout mice after kainic acid injection—suggesting a neuroprotective role for microglial *P2Y12R* in epilepsy [24]. This is probably because the ATP released by hyperactive neurons increases neurons-microglia contact via *P2Y12R* during the seizure-onset phase and in consequence exerts a suppression of neuronal activity via A1 receptors [28,29]. Further study indicated that a *P2Y12R*-dependent mechanism in microglia promoted aberrant neurogenesis and increased immature neuronal projections following seizures, which contributed to the development of epilepsy [26]. If *P2Y12R* is proved to impact on microglia function during epilepsy in basic research, could its single-nucleotide polymorphism be involved in the etiology of epilepsy?

Previous studies demonstrated that the *P2Y12R* rs1491974 might be related to moderate residual platelet reactivity in coronary artery disease, while the *P2Y12R* rs6798347 could be associated with ADP-induced platelet aggregation [30,31]. However, the relationship between these two *P2Y12R* SNPs and epilepsy is unclear. Therefore, our study aimed to investigate the association between *P2Y12R* gene polymorphisms (rs1491974 and rs6798347) and epilepsy.

## Patients and methods

### Study participants

As a case-control study, 200 patients with epilepsy (PWEs) and 50 healthy participants were recruited at the Sichuan Academy of Medical Science and Sichuan Provincial People’s Hospital in China between August 2020 and August 2021. All study protocols were approved by the Ethics Committee of the Sichuan Academy of Medical Science and Sichuan Provincial People’s Hospital. Written informed consent was obtained from either the participants or their guardians. PWEs were diagnosed according to the 2014 International League Against Epilepsy criteria [32]. Those with a history of pseudo-epileptic seizures, as well as those with impaired hepatic and/or renal function, were excluded. The clinical data of patients were collected, including gender, age, disease diagnosis, seizure onset frequency, epilepsy onset, medical history, and imaging examination. Individuals missing the above-mentioned clinical data were excluded from the study. The healthy controls were neurologically normal and had no personal or family history of epilepsy.

### DNA extraction and genotyping

A 5-mL sample of anti-coagulant peripheral blood was taken from each participant. Genomic DNA was extracted from whole blood samples using a Qiagen kit (Qiagen, Hilden, Germany) according to the manufacturer’s recommendations and stored at –80 °C until further use. All SNPs were genotyped using the MassARRAY platform (Agenda Bioscience, San Diego, CA, USA) at CapitalBio (Beijing, China). The primers for PCR amplification and extension were designed using the MassARRAY Assay Design v4.0 software. The PCR cycle program as well as shrimp alkaline phosphatase digestion and extension was performed according to the manufacturer’s protocol. Extension products were desalted and detected using matrix-assisted laser desorption ionization time-of-flight. Finally, the data was processed with TYPER v4.0 software (Agena Bioscience, San Diego, CA, USA).

### Statistical analysis

All statistical analyses were conducted with SPSS v26.0 software (Chicago, IL, USA). Categorical variables of baseline characteristics were performed as proportions and continuous variables as medians with interquartile ranges. Differences in the demographic characteristics between the two groups were analyzed by the non-parametric independent-samples Wilcoxon signed-rank test for continuous variables and the chi-square test for categorical data. The chi-square test was used to assess the deviation from Hardy–Weinberg equilibrium. The chi-square statistics or Fisher’s exact test was used to compare the statistical differences in genotype distributions and allele frequencies between cases and controls. The odds ratio (OR) was calculated with 95% confidence intervals (CIs). Statistical significance was defined as two-tailed *p* < 0.05.

## Results

### Clinical characteristics of the study population

A total of 200 PWEs participated in the study, with 192 of them satisfactorily genotyped for both SNPs. A total of 16 participants were removed from the study due to a lack of clinical data. Therefore, our study included 176 PWEs (85 males, 91 females; median age: 29 years) and 50 healthy controls (22 males, 28 females; median age: 26 years). There was no statistically significant difference between epileptic patients and healthy controls in terms of gender. Table 1 demonstrates the demographic and clinical characteristics of the study population.

**Table 1:** Demographic and clinical characteristics of the study population

Variables	Epileptic patients	Healthy controls	*p* value
	(*n*=176)	(*n*=50)	
Age (years)	29 (24–47)	26 (25–28)	0.001
Gender			
Male	85 (48.3%)	22 (44.0%)	0.591
Female	91 (51.7%)	28 (56.0%)	
Drug treatment			
Monotherapy	111 (63.1%)	-	-
Polytherapy	58 (33.0%)	-	-
NO	7 (3.9%)	-	-
Treatment response			
Drug-resistant	32 (18.2%)	-	-
Drug-responsive	65 (36.9%)	-	-
Undefined	79 (44.9%)	-	-
Neuroimaging			
Abnormal	78 (43.8%)	-	-
Normal	98 (56.2%)	-	-
Epileptic seizure frequencies			
< 2 times/year	65 (36.9%)	-	-
≥ 2 times/year	111 (63.1%)	-	-

### Associations of the P2Y12R gene polymorphisms with epilepsy

Table 2 shows the genotypes or alleles of the two SNPs (rs1491974 and rs6798347) in PWEs and controls. Subsequently, we stratified the groups by gender, neuroimaging, epileptic seizure frequency, and treatment response (Tables 3, 4, 5, and 6). Our results demonstrated that the frequency of the rs1491974 G allele was significantly higher among all patients than in healthy controls (OR = 0.576, 95% CI = 0.368–0.901, *p* = 0.015 for A vs. G). We also found the distribution of the G allele of epileptic patients with negative intracranial imaging was significantly higher than that of the healthy individuals (OR = 0.600, 95% CI = 0.369–0.975, *p* = 0.038 for A vs. G). These results illustrated those individuals with the G allele of rs1491974 G>A might have higher risks for epilepsy. After separating the groups by gender, the differences appeared to be limited to healthy controls and female patients (*p* = 0.004 for genotype; *p* = 0.001 for allele). In female patients, we found the GG genotype frequency was markedly higher than that of the controls (OR = 3.450, 95% CI = 1.204–9.883, *p* = 0.017 for GG vs. AA/AG), indicating that the GG genotype of *P2Y12R* rs1491974 may be closely related to epilepsy susceptibility in females. Subgroup analyses were also conducted stratified for epileptic seizure frequency. We found the homozygous AA and GG genotypes were associated with a lower risk of frequent seizures for patients, while the heterozygous AG genotype was related to a higher risk (OR = 0.476, 95% CI = 0.255–0.890; *p* = 0.019 for AA/GG vs. GG). Additionally, we did not detect a significant association between rs1491974 and rs6798347 with the *P2Y12R* gene and treatment response.

**Table 2:** Genotypic and allelic distribution of the *P2Y12R* gene between all patients and controls

SNP	Genetic model	Genotype/allele	Cases	Controls	OR	95% CI	*p* value
rs1491974	Codominant	AA vs. AG vs. GG	27 (15.3%)/88 (50.0%)/61 (34.7%)	14 (28.0%)/26 (52.0%)/10 (20.0%)	-	-	0.047*
	Allele contrast	A vs. G	142 (40.3%)/210 (59.7%)	54 (54.0%)/46 (46.0%)	0.576	0.368–0.901	0.015*
	Dominant	GG vs. AG+AA	61 (34.7%)/115 (65.3%)	10 (20.0%)/40 (80.0%)	2.122	0.993–4.534	0.049*
	Recessive	AG+GG vs. AA	149 (84.7%)/27 (15.3%)	36 (72.0%)/14 (28.0%)	2.146	1.023–4.503	0.040*
	Overdominant	AA+GG vs. AG	88 (50.0%)/88 (50.0%)	24 (48.0%)/26 (52.0%)	1.083	0.578–2.031	0.803
rs6798347	Codominant	AA vs. AG vs. GG	7 (4.0%)/61 (34.6%)/108 (61.4%)	6 (12.0%)/19 (38.0%)/25 (50.0%)	-	-	0.069
	Allele contrast	A vs. G	75 (21.3%)/277 (78.7%)	31 (31.0%)/69 (69.0%)	0.603	0.367–0.988	0.043*
	Dominant	GG vs. AG+AA	108 (61.4%)/68 (38.6%)	25 (50.0%)/25 (50.0%)	1.588	0.844–2.988	0.150
	Recessive	AG+GG vs. AA	169 (96.0%)/7 (4.0%)	44 (88.0%)/6 (12.0%)	3.292	1.053–10.292	0.032*
	Overdominant	AA+GG vs. AG	115 (65.4%)/61 (34.6%)	31 (62.0%)/19 (38.0%)	1.155	0.603–2.213	0.663

*CI*, confidence interval; *OR*, odds ratio

^*^p<0.05

**Table 3:** Genotypic and allelic distribution of the *P2Y12R* gene between patients with negative intracranial imaging and controls

SNP	Genetic model	Genotype/allele	Cases	Controls	OR	95% CI	*p* value
rs1491974	Codominant	AA vs. AG vs. GG	16 (16.3%)/49 (50.0%)/33 (33.7%)	14 (28.0%)/26 (52.0%)/10 (20.0%)	-	-	0.112
	Allele contrast	A vs. G	81 (41.3%)/115 (58.7%)	54 (54.0%)/46 (46.0%)	0.600	0.369–0.975	0.038*
	Dominant	GG vs. AG+AA	33 (33.7%)/65 (66.3%)	10 (20.0%)/40 (80.0%)	2.031	0.904–4.564	0.083
	Recessive	AG+GG vs. AA	82 (83.7%)/16 (16.3%)	36 (72.0%)/14 (28.0%)	1.993	0.880–4.513	0.095
	Overdominant	AA+GG vs. AG	49 (50.0%)/49 (50.0%)	24 (48.0%)/26 (52.0%)	1.083	0.548–2.142	0.818
rs6798347	Codominant	AA vs. AG vs. GG	3 (3.1%)/39 (39.8%)/56 (57.1%)	6 (12.0%)/19 (38.0%)/25 (50.0%)	-	-	0.096
	Allele contrast	A vs. G	45 (23.0%)/151 (77.0%)	31 (31.0%)/69 (69.0%)	0.663	0.387–1.137	0.134
	Dominant	GG vs. AG+AA	56 (57.1%)/42 (42.9%)	25 (50.0%)/25 (50.0%)	1.333	0.673–2.641	0.409
	Recessive	AG+GG vs. AA	95 (96.9%)/3 (3.1%)	44 (88.0%)/6 (12.0%)	4.318	1.032–18.067	0.062
	Overdominant	AA+GG vs. AG	59 (60.2%)/39 (39.8%)	31 (62.0%)/19 (38.0%)	0.927	0.461–1.867	0.832

*CI*, confidence interval; *OR*, odds ratio

∗*p*<0.05

**Table 4:** Genotypic and allelic distribution of the *P2Y12R* gene between all patients and controls in different genders

SNP	Gender	Genetic model	Genotype/allele	Cases	Controls	OR	95% CI	*p* value
rs1491974	Male	Codominant	AA vs. AG vs. GG	15 (17.6%)/48 (56.5%)/22 (25.9%)	3 (13.7%)/14 (63.6%)/5 (22.7%)	-	-	0.822
		Allele contrast	A vs. G	78 (45.9%)/92 (54.1%)	20 (45.5%)/24 (54.5%)	1.017	0.523–1.980	0.960
		Dominant	GG vs. AG+AA	22 (25.9%)/63 (74.1%)	5 (22.7%)/17 (77.3%)	1.187	0.392–3.599	0.761
		Recessive	AG+GG vs. AA	70 (82.4%)/15 (17.6%)	19 (86.3%)/3 (13.7%)	0.737	0.193–2.812	0.761
		Overdominant	AA+GG vs. AG	37 (43.5%)/48 (56.5%)	8 (36.4%)/14 (63.6%)	1.349	0.512–3.554	0.544
	Female	Codominant	AA vs. AG vs. GG	12 (13.2%)/40 (44.0%)/39 (42.8%)	11 (39.3%)/12 (42.9%)/5 (17.8%)	-	-	0.004*
		Allele contrast	A vs. G	64 (35.2%)/118 (64.8%)	34 (60.7%)/22 (39.3%)	0.351	0.189–0.650	0.001*
		Dominant	GG vs. AG+AA	39 (42.8%)/52 (57.2%)	5 (17.8%)/23 (82.2%)	3.450	1.204–9.883	0.017*
		Recessive	AG+GG vs. AA	79 (86.8%)/12 (13.2%)	17 (60.7%)/11 (39.3%)	4.260	1.612–11.255	0.002*
		Overdominant	AA+GG vs. AG	51 (56.0%)/40 (44.0%)	16 (57.1%)/12 (42.9%)	0.956	0.407–2.249	0.918
rs6798347	Male	Codominant	AA vs. AG vs. GG	2 (2.3%)/31 (36.5%)/52 (61.2%)	1 (4.5%)/10 (45.5%)/11 (50.0%)	-	-	0.450
		Allele contrast	A vs. G	35 (20.6%)/135 (79.4%)	12 (27.3%)/32 (72.7%)	0.691	0.323–1.479	0.340
		Dominant	GG vs. AG+AA	52 (61.2%)/33 (38.8%)	11 (50.0%)/11 (50.0%)	1.576	0.614–4.045	0.342
		Recessive	AG+GG vs. AA	83 (97.7%)/2 (2.3%)	21 (95.5%)/1 (4.5%)	1.976	0.171–22.849	0.502
		Overdominant	AA+GG vs. AG	54 (63.5%)/31 (36.5%)	12 (54.5%)/10 (45.5%)	1.452	0.562–3.747	0.440
	Female	Codominant	AA vs. AG vs. GG	5 (5.5%)/30 (33.0%)/56 (61.5%)	5 (17.9%)/9 (32.1%)/14 (50.0%)	-	-	0.112
		Allele contrast	A vs. G	40 (22.0%)/142 (78.0%)	19 (33.9%)/37 (66.1%)	0.549	0.285–1.056	0.070
		Dominant	GG vs. AG+AA	56 (61.5%)/35 (38.5%)	14 (50.0%)/14 (50.0%)	1.600	0.682–3.753	0.278
		Recessive	AG+GG vs. AA	86 (94.5%)/5 (5.5%)	23 (82.1%)/5 (17.9%)	3.739	0.997–14.028	0.039*
		Overdominant	AA+GG vs. AG	61 (67.0%)/30 (33.0%)	19 (67.9%)/9 (32.1%)	0.963	0.389–2.382	0.935

*CI*, confidence interval; *OR*, odds ratio

∗*p*<0.05

**Table 5:** Genotypic and allelic distribution of the *P2Y12R* gene between patients with epileptic seizure frequencies < 2 times/year and patients with epileptic seizure frequencies ≥ 2 times/year

SNP	Genetic model	Genotype/allele	Epileptic seizure frequencies < 2 times/year	Epileptic seizure frequencies ≥ 2 times/year	OR	95% CI	*p* value
rs1491974	Codominant	AA vs. AG vs. GG	6 (9.2%)/40 (61.6%)/19 (29.2%)	21 (18.9%)/48 (43.2%)/42 (37.9%)	-	-	0.047*
	Allele contrast	A vs. G	52 (40.0%)/78 (60.0%)	90 (40.5%)/132 (59.5%)	0.978	0.629–1.520	0.921
	Dominant	GG vs. AG+AA	19 (29.2%)/46 (70.8%)	42 (37.9%)/69 (62.1%)	0.679	0.351–1.301	0.247
	Recessive	AG+GG vs. AA	59 (90.8%)/6 (9.2%)	90 (81.1%)/21 (18.9%)	2.294	0.874–6.022	0.085
	Overdominant	AA+GG vs. AG	25 (38.4%)/40 (61.6%)	63 (56.8%)/48 (43.2%)	0.476	0.255–0.890	0.019*
rs6798347	Codominant	AA vs. AG vs. GG	1 (1.5%)/25 (38.5%)/39 (60.0%)	6 (5.4%)/36 (32.4%)/69 (62.2%)	-	-	0.374
	Allele contrast	A vs. G	27 (20.8%)/103 (79.2%)	48 (21.6%)/174 (78.4%)	0.950	0.559–1.616	0.850
	Dominant	GG vs. AG+AA	39 (60.0%)/26 (40.0%)	69 (62.2%)/42 (37.8%)	0.913	0.488–1.710	0.776
	Recessive	AG+GG vs. AA	64 (98.5%)/1 (1.5%)	105 (94.6%)/6 (5.4%)	3.657	0.430–31.074	0.205
	Overdominant	AA+GG vs. AG	40 (61.5%)/25 (38.5%)	75 (67.6%)/36 (32.4%)	0.768	0.406–1.454	0.417

*CI*, confidence interval; *OR*, odds ratio

∗*p*<0.05

**Table 6:** Genotypic and allelic distribution of the *P2Y12R* gene between drug-resistant patients and drug-responsive patients

SNP	Genetic model	Genotype/allele	Drug-resistant patients	Drug-responsive patients	*p* value
rs1491974	Codominant	AA vs. AG vs. GG	6 (18.8%)/13 (40.6%)/13 (40.6%)	10 (15.4%)/38 (58.4%)/17 (26.2%)	0.235
	Allele contrast	A vs. G	25 (39.1%)/39 (60.9%)	58 (44.6%)/72 (55.4%)	0.462
rs6798347	Codominant	AA vs. AG vs. GG	1 (3.1%)/10 (31.3%)/21 (65.6%)	4 (6.2%)/28 (43.1%)/33 (50.7%)	0.447
	Allele contrast	A vs. G	12 (18.8%)/52 (81.2%)	36 (27.7%)/94 (72.3%)	0.175

∗*p*<0.05

For the *P2Y12R* rs6798347 G>A polymorphism, the frequency of the G allele was substantially greater in all patients than in the healthy controls (OR = 0.603, 95% CI = 0.367–0.988, *p* = 0.043 for A vs. G). Comparing PWEs with negative intracranial imaging and healthy controls, there were no variations in allelic or genotypic distribution. In addition, after grouping by gender, epileptic seizure frequency, and treatment response, we discovered that there were no significant differences between PWEs and controls.

## Discussion

The results of the present study showed a significant difference in the G allele frequency of *P2Y12R* rs1491974 and rs6798347 polymorphisms between PWEs and healthy participants, indicating that *P2Y12R* genetic variability might be associated with epilepsy. Consistent with our result, animal studies have shown that *P2Y12R*-deficient mice had exacerbated behavioral seizures after intraperitoneal kainic acid injection and the percentage of mice showing seizures increased by inactivating the *P2Y12R* gene [24],[26],[33]. From these findings, *P2Y12R* was presented to play a role in epilepsy.

The P2Y12 receptor was originally thought to be exclusively expressed on platelets [34]. Thus, a great deal of previous research about *P2Y12R* gene polymorphisms focused on platelet aggregation or pharmacological response to anti-platelet drugs [35-38]. Nevertheless, recent studies demonstrated that the *P2Y12R* is expressed and functional in microglial cells [39,40],Gómez et al., 2021,[41]. Microglial *P2Y12R* detects the synaptic release of ATP after neuronal activity and controls chemotaxis and motility of microglia, which are involved in microglia-mediated suppression of neuronal activities [14, 15, 28, 29, 42]. As mentioned, the findings of Badimon et al. in *P2Y12R*-inactivated mice suggest microglial *P2Y12R* may have a hyperactivity-limiting role in epilepsy [33]. However, in contrast, another research group found that P2Y12 receptor may play hyperactivity-promoting roles in epileptogenesis after the initial seizure, since a *P2Y12R* deficiency in mice limits the extent of neurogenesis and sprouting that are thought to promote spontaneous recurring seizures [26]. Moreover, the extracellular ADP promoted the activation of NLRP3 inflammasomes and the release of IL-1β and IL-6 via the P2Y12 receptor [43]. These inflammatory cytokines could promote neurogenesis and the excitability of neurons, which may lead to the development of epilepsy [44,45]. Together, *P2Y12R* SNPs may impact on microglia function during epilepsy. To prove our hypothesis, further studies should be performed to verify the association of microglia and *P2Y12R* SNPs.

Several epidemiologic studies have indicated that epilepsy was more common in males than in females [46-49]. Interestingly, our findings indicated the G allele or GG genotype of rs1491974 (G>A) was more predominant in female patients with epilepsy than in their control counterparts, whereas no significant differences were shown in males. A potential explanation for the disparity may involve the differences in endogenous sex hormones, such as androgen, estrogen, and progesterone, as well as their metabolites, which play a vital role in brain network construction and neuro-immune system activity [50]. This agrees with the findings of Wang et al. [51] and [52], who discovered that gender-specific incidence was higher for male partial seizures than for females in NLRP1 SNPs (rs878329, G>C) and NRG1 SNPs (rs35753505, T>C). We also observed an increased seizure frequency in individuals with the AG genotype of *P2Y12R* rs1491974. Overall, our findings suggest that *P2Y12R* gene variants influence some characteristics of expression in epilepsy patients. In addition, though our results suggest that *P2Y12R* gene polymorphisms do not correlate with the response to antiepileptic drugs, this still requires further investigation.

Our study is not without limitations. First, we only analyzed the population in southern China and lack representation from other regions of the country. Future studies should involve patients from the greater China region. Second, the SNPs of *P2Y12R* have not been reported in epilepsy. Hence, the discussion concerning the SNPs of *P2Y12R* is limited. Further validation and studies are necessary to confirm the relationship between *P2Y12R* and epilepsy. Third, this study was confined to the association of SNPs with epilepsy and lacked specific epileptic subtypes due to the limited sample size. Thus, more research is warranted to improve our understanding of the association between *P2Y12R* and the pathophysiology of epilepsy.

## Data Availability

The datasets generated and/or analyzed during the current study are available from the corresponding author on reasonable request.
